# Prevention of Chronic kidney disease (CKD) in children

**DOI:** 10.1186/1824-7288-41-S2-A56

**Published:** 2015-09-30

**Authors:** Carmine Pecoraro

**Affiliations:** 1 Department of Nephrology and Urology, Unit of Nephrology and Dialysis, Children Hospital Santobono-Pausilipon, Naples, Italy

## 

CKD is recognized worldwide as an important problem in children. The overt stage of CKD is end-stage renal disease, which is merely the tip of the iceberg of a large number of “covert less severe diseases." CKD represents a developing process that is initiated by various causes, all with the common end result of persistent and usually progressive damage of varying severity to the kidneys. It is characterized by the common histopathological end point of glomerulosclerosis, tubulointerstitial fibrosis and tubular atrophy, irrespective of the underlying etiology of kidney disease. The aim of the presentation is to identify the clinical and pathological conditions that cause renal damage and suggest measures for its prevention. CKD is defined by the following criteria: a) Kidney damage for ≥3 months, as defined by structural or functional abnormalities of the kidney with or without decreased glomerular filtration rate (GFR) manifested by one or more of the following features: abnormalities in the composition of the blood or urine, abnormalities in imaging tests, abnormalities on kidney biopsy; b) GFR <60 ml/min/1.73 m^2^ for ≥3 months with or without the other above-mentioned signs of kidney damage. CKD has been classified into five stages for the purpose of prevention, early identification of renal damage and institution of preventative measures for progression of primary damage and appropriate guidelines for instituting management for prevention of complications in severe CKD [1]. Pediatricians have the opportunity to screen at-risk patients, identify affected patients, prevent renal damage and ameliorate the impact of CKD by initiating early therapy and monitoring disease progression. The prevention of CKD constitutes three important aspects.

*Primary prevention* aims to eliminate or reduce exposure to factors that cause renal disease.

*Secondary prevention* in which the prevention of the progression of renal damage from stage 1 to stage 5 is carried out by introducing appropriate measures at various stages of CKD.

*Tertiary prevention* strategies are focussed on the reduction or delay of long-term complications, impairments or disabilities in established disease, requiring renal replacement therapy (RRT).

Meanwhile, in adults, the primary preventative measures are limited to few conditions, such as diabetes mellitus, atherosclerosis and hypertension, in children there is a wide range of conditions that can start even before birth; if not recognized earlier, these conditions can cause renal damage and progress to CKD in childhood, adolescence or later in the adult life.

## References

[B1] Hogg RJ Furth S Lemley KV Portman R Schwartz GJ Coresh J National Kidney Foundation's Kidney Disease Outcomes Quality Initiative Clinical Practice Guidelines for Chronic Kidney Disease in Children and Adolescents: Evaluation, classification and stratification Pediatrics 2003 111 6 Pt 1 1416 1421 10.1542/peds.111.6.1416 12777562

